# Monthly Summary

**Published:** 1871-10

**Authors:** 


### MONTHLY SUMMARY.

*Spoon Feeding.*—Dr. Furst has published a pamphlet on this subject in Leipzig, wherein he gives good advice to mothers. To judge of the success of this kind of feeding, the author gives the following criteria:—At birth the child weighs about from six pounds and a quarter to eight pounds, the first for girls, the second for boys. During the first three days the infant loses about four ounces, but recovers its original weight about the ninth day. The child should, in the first four months increase from 300 to 375 grains per diem, and later only from 150 to

225 grains. At twelve months old a boy should weigh twenty pounds, and a girl about eighteen pounds and a half. If the child is far below the averages just mentioned, the assistance of a medical man should be requested.—*Med. and Surg. Rep.*

---

*Sulphate of Nickel in Neuralgia.*—A case of obstinate neuralgia is related, which was cured by sulphate of nickel, in doses of half a grain, three times a day. At the end of a week one grain was given. Its relative action was speedily manifested in reducing the pulse and procuring sleep; all symptoms of the paroxysm disappeared.—*Oregon Med. and Surg. Rep.*

---

*Disinfecting Cotton.*—Dr. Fresenius possesses a method for applying permanganate of potassa which seems to overcome many of the difficulties hitherto felt in practice, and this consists in saturating gun cotton with a solution of the permanganate of potash. The gun cotton is not decomposed by the manganese salt, as ordinary cotton is, but serves to expose and keep the greatest amount of surface for the action of the disinfectant Bandages of the gun cotton thus saturated with permanganate of potash can be readily applied, and in cases of open wounds, cancers, &c. must prove very acceptable to surgeons.—*N. Y. Med. Gazette.*

---

*Innocent and Morbid Growths.*—J. N. Danforth, M. D., Chicago, Illinois, (*Chicago Medical Journal,*) in a paper on the "Microscopic Appearances of Cancer-cells," lays down the following simple rules for drawing the distinction between innocent and morbid growths: Whenever a description of *one* of the cells of a microscopic specimen is a description of *all* of its cells, the chances are as ten to one that it is *not* cancer; whenever on the other hand, the cells of such a specimen are so varied in form and size that philology, and ingenuity, and imagination, and the most unflinching resolution, combined, utterly fail to accomplish the task of describing them, the chances are as ten to one that the specimen is from a malignant growth, whatever may be its name or location.—*Medical Record.*

*Epithelioma Removed by Bromine.*—Dr. Wynn Williams showed to the Obstetrical Society of London, "a patient, nearly the whole of whose lower lip had been removed for epithelioma eighteen months previously. The disease shortly appearing in the cicatrix, the growth was succesfully treated by two injections of bromine, twenty drops to a drachm of spirit. There was no appearance of disease at present."—*Lancet.*

---

*A Bullet Makes Eight Wounds.*—Sandford Moore, B. A., Assistant-Surgeon Fourth Dragoon Guards, in an interesting paper on "The Gunshot Wounds of the war," published in the *London Lancet* for July, 1871, says: "The bullet-wounds demanded the surgeon's attention, not alone from the injury inflicted on the parts, but from the eccentric course sometimes taken by the ball, One bullet dropped down a man's trowsers and fell into his boot, having previously passed through both thighs scrotum, prepuce, making in all eight wounds caused by this one ball. Another I have seen where the ball lodged in and was subsequently extracted from the right iliac region, having before this passed through the left elbow-joint and across the abdomen. And a third exemplifying the erratic passage of the ball: A soldier received a bullet-wound of the left thigh; and this was the only one which could be discovered about this extremity, or, in short, the only wound: nevertheless a bullet was found lodged in his right thigh, a short way above his knee, and was subsequently extracted. Where was the wound of entrance? I presume it was on the opposite thigh, and the ball must then have travelled round by the pelvis to where it was found lodged."—*Nashville Journal of Medicine and Surgery.*

---

*Posture of the Head in Sleeping.*—It is often a question among people that are unacquainted with anatomy and physiology, whether lying with the head exalted or on a level with the body, is the more unwholesome. Most, consulting their own ease on this point, argue in favor of that which they prefer. Now, although many delight in bolstering up their heads at night, and sleep soundly without injury, yet we declare it to be a dangerous habit. The vessels in which the blood passes from the heart to the head are always lessened in their cavities when the head is resting in bed higher than the body; therefore, in all diseases attended with fever, the head should be pretty nearly on a level with the body; and people ought to accustom themselves to sleep thus, and avoid danger.—*Home and Health.*

---

*A Test for Purity of Hydrate of Chloral.*—The Editor of the *Boston Journal of Chemistry*, in answer to a correspondent, states that when a fragment of pure hydrate of chloral is placed in a test-tube or wine-glass with a little water, and a few drops of liquor potassæ allowed to fall upon it, no discoloration takes place, and there is evolved pure chloroform, detected by the odor. If the specimen is impure, a dark brown reaction will result, and the odor evolved will be unpleasant.—*Clinical Record.*

---

*Permanganate of Potash Injections.*—Permanganate of potash, of the strength of five grains to the ounce of water, is a valuable injection of gonorrhœa and gleet. The strength may be increased up to fifteen grains. It should be used four times a day. As the permanganate loses its virtue by admixture with any extraneous matter, or exposure, it is advisable to mix the injection immediately before using it.—*Dr. T Ward. Braithwaite's Retrospect.*

---

*Rare Form of Hare-lip.*—Thomas Ogden æt. 4 months, has a fissure of the upper lip, situated to the right of the median line. Such occurrence is rare. Hare-lip may vary in degree from a slight fissure extending half way to the nostril extending entirely through the lip, the alveolar process of the superior maxillary and the hard and soft palates. Double hare-lip is not of unfrequent occurrence. The affection is always congenital and always occupies the upper lip. Dr. Gross narrated a case of a boy brought to him in Kentucky, where the fissure extended into the cheek. The edges were pared and brought together with pins and the twisted suture, and the boy made a good recovery.

Do not operate upon children of too tender an age. As a rule, wait until dentition commences; for before that period the blood is lacking in that plastic material which so thoroughly glues the edges of the wound together.

The steps of the operation consisted in first separating the lip from the gum. An assistant then grasped the upper lip on either side of the fissure, so as to control hemorrhage occurring from the severed coronary arteries, and the margins pared. Two hare-lip pins were then inserted in such a manner that one-third of the pin was behind and two-thirds in front of the lip, thus making them embrace the coronary artery. At the cutaneous margin of the lip an interrupted suture was employed, so as to bring the parts more accurately in apposition, and thus lessen the chances of deformity. The upper pin will be allowed to remain in 48 hours. The lower pin three days, and the suture a day or two later.—*Med. and Surg. Reporter.*

